# Insight into noncanonical small noncoding RNAs in Influenza A virus infection

**DOI:** 10.1016/j.virusres.2024.199474

**Published:** 2024-09-27

**Authors:** Eun-A Ko, Tong Zhou, Jae-Hong Ko

**Affiliations:** a Department of Physiology, College of Medicine, Jeju National University, Jeju 63243, South Korea; b Department of Physiology and Cell Biology, University of Nevada, Reno School of Medicine, Reno, NV 89557, USA; c Department of Physiology, College of Medicine, Chung-Ang University, Seoul 06974, South Korea

**Keywords:** Small noncoding RNAs, Influenza A virus, tsRNAs, rsRNAs, ysRNAs

## Abstract

Influenza A virus (IAV) induces acute respiratory infections in birds and various mammals, including humans, and presents a significant global public health concern, with considerable economic consequences. Recently, researchers have shown keen interest in noncanonical small noncoding RNAs (sncRNAs) as carriers of epigenetic information, including tRNA-derived small RNAs (tsRNAs), rRNA-derived small RNA (rsRNAs), and Y RNA-derived small RNAs (ysRNAs). Particularly, tsRNAs and rsRNAs are detected in diverse species and demonstrate evolutionary conservation. We analyzed sncRNAs sequencing data in the pulmonary tissue of two genetically distinct mouse strains, C57BL/6J and DBA/2J, to explore strain-specific variations of sncRNAs in response to IAV infection. We systematically compiled information on noncanonical sncRNAs in these two strains and investigated the tsRNAs/rsRNAs/ysRNAs profiles influenced by IAV infection. Specifically, four noncanonical sncRNA families, including rsRNA-12S, GtsRNA-Arg-CCT, GtsRNA-Arg-TCT, and GtsRNA-Lys-TTT, exhibited upregulation upon IAV infection. Notably, DBA/2J mice showed earlier systemic differential expression of noncanonical sncRNAs after IAV infection compared to C57BL/6J mice. Additionally, our study revealed a strain-specific biogenesis of MtsRNAs in response to IAV infection. Also, distinct co-expression patterns of MtsRNAs were observed between C57BL/6J and DBA/2J mice, with DBA/2J mice showing broader positive co-expression of MtsRNAs with various sncRNA families compared to C57BL/6J mice. Our study provides a novel insight into noncanonical sncRNAs and their implications in IAV pathology and mouse strain specificity.

## Highlights

• Different expressions of ts/rs/ys RNAs following IAV infection between DBA/2J and C57BL/6J mouse strains.
• rsRNA-12S, GtsRNA-Arg-CCT, GtsRNA-Arg-TCT, and GtsRNA-Lys-TTT exhibited upregulation upon IAV infection.
• The susceptibility to IAV infection is higher in DBA/2J compared to C57BL/6J.

## 1 Introduction

Influenza A virus (IAV) is an infectious respiratory virus that can infect various hosts, demonstrating a high degree of transmissibility and a capacity for rapid mutation. IAV contributes significantly to the global economic burden (Cheng et al., 2024; de Courville et al., 2022; Scheibner et al., 2023; Shah et al., 2024). During the early stages of IAV infection, the innate immune system is activated to protect the host against the invading virus (Casalino-Matsuda et al., 2024; Mettelman et al., 2023). Besides innate immunity, earlier research has explored that noncoding RNAs (ncRNAs) play a role in regulating host response to viral infection (Behnia and Bradfute, 2023; Liao et al., 2021; Rajput et al., 2020). ncRNAs are functional RNAs not involved in direct protein synthesis. Generally, ncRNAs can be classified into two groups; small noncoding RNAs (sncRNAs) and long noncoding RNAs. In addition to the well-explored sncRNA classes, such as microRNAs (miRNAs) and Piwi-interacting RNA (piRNAs), the analysis of sncRNA sequencing (sncRNA-seq) data discovered the expanding universe of noncanonical sncRNAs (Shi et al., 2021, 2022). These also include tRNA-derived small RNAs (tsRNAs, also known as tRNA-derived fragments, tRFs), rRNA-derived small RNA (rsRNAs), and Y RNA-derived small RNAs (ysRNAs). Particularly, tsRNAs and rsRNAs have been identified across various species and exhibit evolutionary conservation (Chen et al., 2021). Additionally, the broad range of biological functions associated with noncanonical sncRNAs and their substantial connections to diseases are currently driving the forefront of sncRNA research (Chen et al., 2023; Gong et al., 2022; Hernandez et al., 2023; Liu et al., 2023). Noncanonical sncRNAs, alongside the harbored RNA modifications, potentially serve as carriers of epigenetic information and play a substantial role in transgenerational/intergenerational epigenetic inheritance (Chen et al., 2016; Liu et al., 2023; Zhang et al., 2018). Notably, ysRNAs are exclusively present within mature sperm heads, serving as carriers for the transmission and alteration of epigenetic information that can be passed on through generations (Elzer et al., 2023). tsRNAs and rsRNAs are associated with various epigenetic factors in the reproductive system. For example, changes in sperm tsRNAs and rsRNAs occur in response to challenges such as high-fat or high-sugar diets, potentially influencing sperm functions and contributing to epigenetic inheritance of paternally acquired metabolic disorders (Chen et al., 2016; Natt et al., 2019; Zhang et al., 2018).

Despite the growing interest in host sncRNAs and genetic susceptibility to IAV, only a few studies have identified changes in sncRNAs, such as miRNAs and small nucleolar RNAs, in viral infections (Preusse et al., 2017; Yin et al., 2023; Zhuravlev et al., 2022). Specifically, the distinctive expression of miRNA before and during IAV infection was observed between C57BL/6J and DBA/2J mice, with the DBA/2J strain demonstrating greater susceptibility to IAV infection (Preusse et al., 2017). The diverse susceptibility to IAV infection correlates with transcriptomic alterations shaped by genetic background (Wilk et al., 2015). Consequently, the mouse model represents an excellent tool for examining viral virulence factors and genetic host factors playing a role in disease progression and outcomes.

In this study, we utilized sncRNA-seq data from two inbred mouse strains (Preusse et al., 2017), DBA/2J and C57BL/6J, in the pulmonary tissue to identify strain-specific variations in host sncRNAs in response to IAV infection. We employed the computational framework, *SPORTS*, specifically designed for annotating and profiling noncanonical sncRNAs (Shi et al., 2018). Our study provides a novel insight into noncanonical sncRNAs and their implications in IAV pathology and mouse strain specificity.

## 2 Methods

### 2.1 Dataset

The sncRNA-seq data of this study were obtained from the Gene Expression Omnibus (GEO) with an accession of GSE89064 (Preusse et al., 2017). In total, 63 lung samples from C57BL/6J mice and 62 lung samples from DAB/2J mice were investigated. For the C57BL/6J mice, 5 untreated samples at 0 h, 4/5/5/5/5/4 post mock treatment samples at 6 h/12 h/18 h/24 h/48 h/120 h, and 5/5/5/5/5/5 post IAV (H1N1) infection samples at 6 h/12 h/18 h/24 h/48 h/120 h were included. For the DAB/2J mice, 4 untreated samples at 0 h, 4/5/5/5/5/5 post mock treatment samples at 6 h/12 h/18 h/24 h/48 h/120 h, and 5/5/4/5/5/5 post IAV infection samples at 6 h/12 h/18 h/24 h/48 h/120 h were included.

### 2.2 Preprocessing of the sncRNA-seq data

The *SPORTS* tool (with mismatch tolerance of one) (Shi et al., 2018) was applied to profile the noncanonical sncRNAs, i.e., tsRNAs, rsRNAs, and ysRNAs from the sncRNA-seq data. Reads per million (RPM) was used to measure the abundance of sncRNAs. Quantification of tsRNAs/rsRNAs/ysRNAs was presented at two levels: (i) expression of the individual tsRNA/rsRNA/ysRNA species (i.e., unique sncRNA sequences) and (ii) abundance of the noncanonical sncRNA families (e.g., rsRNA-12S), which was an aggregation of the sncRNA species from the same family (e.g., all the rsRNA species coming from the same parental RNA rRNA-12S).

### 2.3 Data analyses

The data analyses presented in this study were performed using the R programming platform, including *t*-test, two-way ANOVA, *Spearman*’s rank correlation test, principal component analysis, and hierarchical clustering. The *Benjamini-Hochberg* method was applied for *P*-value correction. To identify the differentially expressed noncanonical sncRNAs between the mock treated and IAV infected mice, the *edgeR* tool was applied, using the *TMM* algorithm for read count normalization and likelihood ratio test for differential expression analysis. The sncRNA species with false discovery rate < 5 % and at least two-fold change in expression were defined as differentially expressed.

## 3 Results

### 3.1 Comparison in noncanonical sncRNA profiles between the C57BL/6J and DBA/2J and mouse strains

**Fig. 1:**
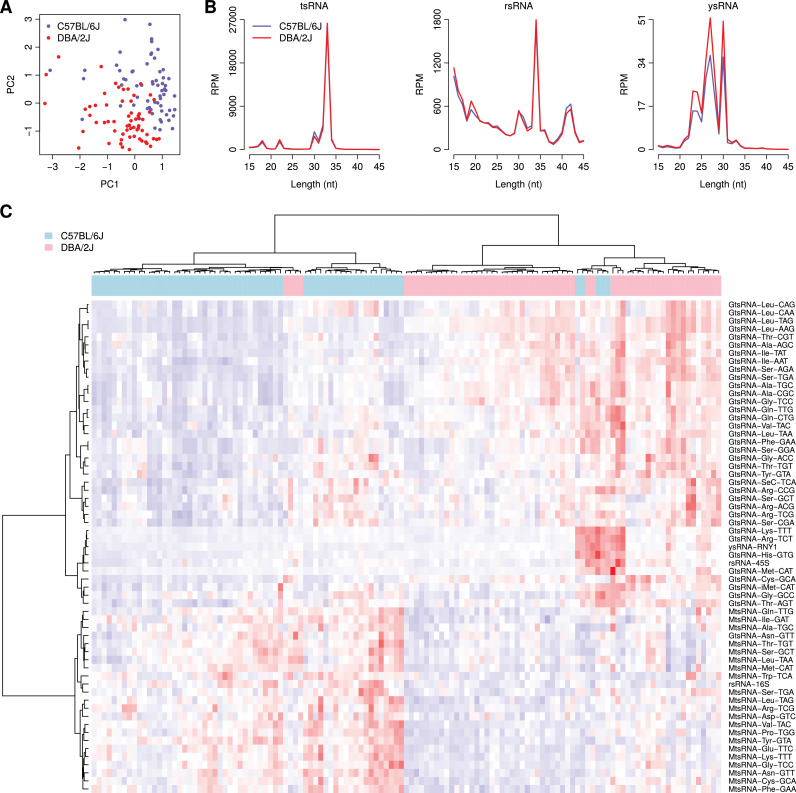
Comparison in noncanonical sncRNA profiles between the two mouse stains. (A) Principal component analysis on the expression of noncanonical sncRNA families. PC1: the first principal component; PC2: the second principal component. (B) Comparison in tsRNA/rsRNA/ysRNA length distribution between the two mouse strains. (C) Expression heatmap of the differentially expressed tsRNA/rsRNA/ysRNA families between the two mouse strains. Red means relatively higher expression while blue denotes lower expression.

In our dataset, there were in total 63 and 62 samples from the C57BL/6J and DBA/2J strains, respectively. We first applied principal component analysis to examine the global difference in the expression pattern of the individual noncanonical sncRNA families between the two strains. Fig. 1A demonstrated that there was a clear shift in the first principal component (PC1) and the second principal component (PC2) between the C57BL/6J and DBA/2J strains (*t*-test: *P* = 3.6 × 10^–7^ for PC1 and *P* = 2.7 × 10^–11^ for PC2), which suggests a global deviation in noncanonical sncRNA expression between the two strains. We next investigated the length distribution of tsRNAs/rsRNAs/ysRNAs in the two strains. A similar length distribution pattern was observed between the two strains: both the C57BL/6J and DBA/2J strains showed a peak for tsRNAs with length of 33 nts; for rsRNAs, the highest peak showed at the length of 34 nts; for ysRNAs, there were two peaks at the length of 27 nts and 30 nts (Fig. 1B). All these results suggest that even though there is a global shift in noncanonical sncRNA expression between the two strains, the mechanism of biogenesis of tsRNAs/rsRNAs/ysRNAs is largely analogous between the C57BL/6J and DBA/2J mice. We further applied two-way ANOVA controlling for treatment to identify the differentially expressed noncanonical sncRNAs between the two mouse strains. In total, 61 tsRNA/rsRNA/ysRNA families (adjusted *P* < 0.05) were identified (Fig. 1C). Interestingly, we observed a systematic upregulation of nuclear genome encoded tsRNAs (GtsRNAs) and downregulation of mitochondrial tsRNAs (MtsRNAs) in the DBA/2J mice relative to the C57BL/6J mice (Fig. 1C).

### 3.2 Time-dependent expression of noncanonical sncRNAs after IAV infection

**Fig. 2:**
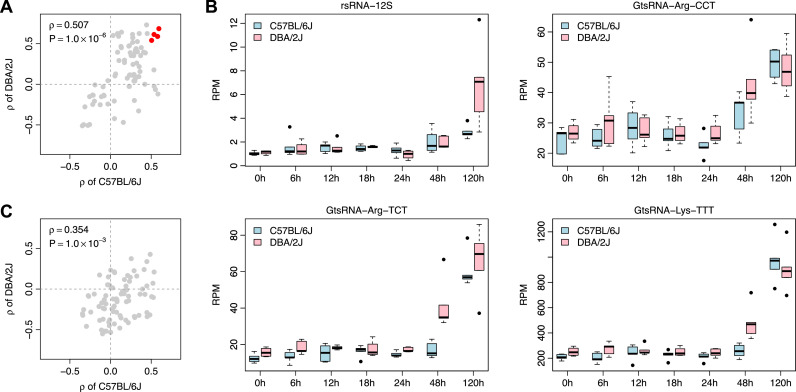
The common differential expressed noncanonical sncRNA families between the two strains. (A) Comparison in the relationship between the expression of noncanonical sncRNA families and IAV post infection hours. *Spearman*’s rank correlation test was used to measure the relationship between the expression of tsRNA/rsRNA/ysRNA families and the infection hours for the C57BL/6J and DBA/2J strains, respectively. Four sncRNA families were identified to be commonly upregulated with the infection hours, which were highlighted in red. (B) Boxplots showing the expression of the sncRNA families highlighted in panel A in response to IAV infection. (C) Comparison in the relationship between the expression of noncanonical sncRNA families and hours of post mock treatment. *Spearman*’s rank correlation test was used to measure the relationship for the C57BL/6J and DBA/2J strains, respectively. No sncRNA family was identified to be commonly upregulated with the mock treatment.

We first identified the tsRNAs/rsRNAs/ysRNA families that exhibited a monotonic time-dependent expression after IAV infection using *Spearman*’s rank correlation test. The expression of one rsRNA family and seven GtsRNA families (adjusted *P* < 0.05) were found to be positively correlated with IAV post infection hours in the C57BL/6J mice (Supplementary Table S1). In comparison, in the DBA/2J mice, the expression of five rsRNA families, two ysRNA families, 16 GtsRNA families, and one MtsRNA family (adjusted *P* < 0.05) were found to be positively correlated with IAV post infection hours, as well as one GtsRNA family and five MtsRNA families being negatively correlated with IAV infection hours (adjusted *P* < 0.05) (Supplementary Table S2). Not surprisingly, the correlation coefficient (*ρ*) in the above analysis is positively correlated between the C57BL/6J and DBA/2J strains (Fig. 2A). Four sncRNA families were identified to be commonly upregulated with post infection hours, including rsRNA-12S, GtsRNA-Arg-CCT, GtsRNA-Arg-TCT, and GtsRNA-Lys-TTT (Fig. 2A and B). Particularly, the upregulation of these sncRNAs appeared to be onset from 48 h in the DBA/2J mice, while the response to infection is generally delayed in the C57BL/6J mice (Fig. 2B). All these results suggest a common pattern in the response to IAV infection between the two strains, although the susceptible DBA/2J mice exhibit increased noncanonical sncRNA levels at earlier time points compared to the IAV-resistant C57BL/6J mice. We next investigated the relationship between the expression of noncanonical sncRNAs and mock treatment. However, no tsRNA/rsRNA/ysRNA family was found to be correlated with post treatment hours in the C57BL/6J mice, while only one GtsRNA family and four MtsRNA families (adjusted *P* < 0.05) were found to be negatively correlated with post treatment hours in the DBA/2J mice (Supplementary Table S3). Also, compared with IAV infection, a weaker correlation in *ρ* between the C57BL/6J and DBA/2J strains was observed for the mock treatment (Fig. 2C).

### 3.3 Differential expression between mock treatment and IAV infection

**Fig. 3:**
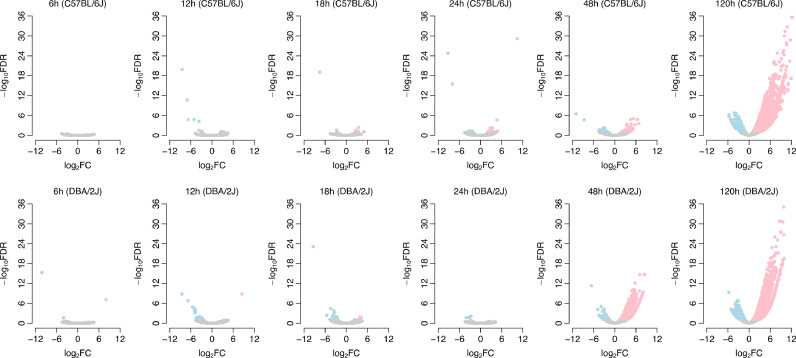
Volcano plots of the differentially expressed sncRNA species between the mock and infection treatments at the individual time points. Each dot represents one sncRNA species. Pink dots indicate the upregulated sncRNA species, while light blue dots indicate the downregulated sncRNA species. The sncRNA species with false discovery rate < 5 % and fold change in expression > 2 were defined as differentially expressed.

**Fig. 4:**
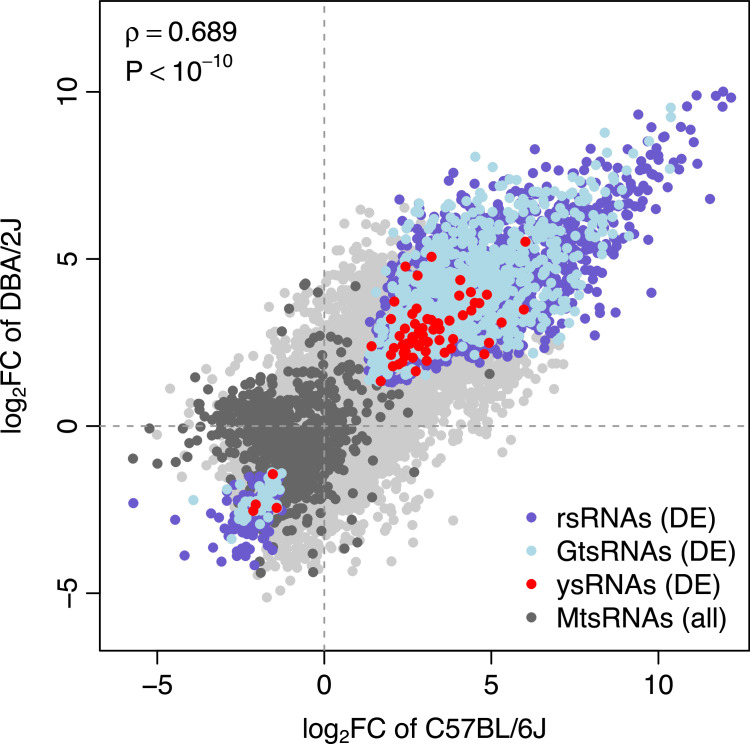
Correlation in log_2_-transformed fold changes between the C57BL/6J and DBA/2J strains. The log_2_*FC* values were computed between the mock and infection treatments at 120 h. The correlation in log_2_*FC* between the two mouse strains was calculated by *Spearman*’s rank correlation test. MtsRNA species show the least accordance in differential expression patterns between the two strains. DE: differentially expressed in both strains.

To investigate the differential expression between mock treatment and IAV infection, we zoomed into the tsRNA/rsRNA/ysRNA species at each time point. Volcano plots demonstrated that there were very few differentially expressed tsRNA/rsRNA/ysRNA species between mock treatment and IAV infection at the earlier time points, i.e., 6 h, 12 h, 18 h, and 24 h (Fig. 3). At 48 h, more differentially expressed sncRNAs were identified in the DBA/2J mice (1462 upregulated and 121 downregulated) compared to the C57BL/6J mice (32 upregulated and 16 downregulated) (Fig. 3), which further supports the observation that the susceptible DBA/2J mice exhibit increased noncanonical sncRNA levels at earlier time points compared to the IAV-resistant C57BL/6J mice. In comparison, at 120 h, the magnitude of differential expression is highly compatible between the two strains (4052 upregulated and 632 downregulated in the C57BL/6J mice while 3932 upregulated and 1102 downregulated in the DBA/2J mice) (Fig. 3). To investigate the similarity in the differential expression pattern between the two strains, we calculated the log_2_-transformed fold changes (log_2_*FC*) for each sncRNA species between the mock treated and IAV infected mice at 120 h. A significant positive correlation in log_2_*FC* was observed between the C57BL/6J and DBA/2J mice (Fig. 4), which suggests that both the strains share a large proportion of differentially expressed tsRNAs/rsRNAs/ysRNAs that exhibit a common response to IAV infection. Briefly, 1408 rsRNA species, 830 GtsRNA species, and 61 ysRNA species were commonly upregulated, while 124 rsRNA species, 43 GtsRNA species, and 4 ysRNA species were commonly downregulated in the IAV infected C57BL/6J and DBA/2J mice (Fig. 4). However, we only identified 5 MtsRNA species that are commonly differentially expressed in both strains (Fig. 4). Indeed, a substantial difference in the number of differentially expressed MtsRNAs was observed for both the 48 h and 120 h time points between the C57BL/6J and DBA/2J mice (Supplementary Fig. S1). We further investigated the correlation in log_2_*FC* at 120 h between the two strains for the individual sncRNA categories separately. A strong positive correlation was observed for GtsRNAs, rsRNAs, and ysRNAs (*Spearman*’s rank correlation test: *ρ* = 0.678 and *P* < 10^–10^ for GtsRNAs, *ρ* = 0.710 and *P* < 10^–10^ for rsRNAs, and *ρ* = 0.656 and *P* < 10^–10^ for ysRNAs), while there was no significant correlation for MtsRNAs (*Spearman*’s rank correlation test: *ρ* = 0.044 and *P* = 0.235). All these results suggest a strain-specific biogenesis mechanism of MtsRNAs upon IAV infection.

### 3.4 Strain-specific co-expression of MtsRNAs

**Fig. 5:**
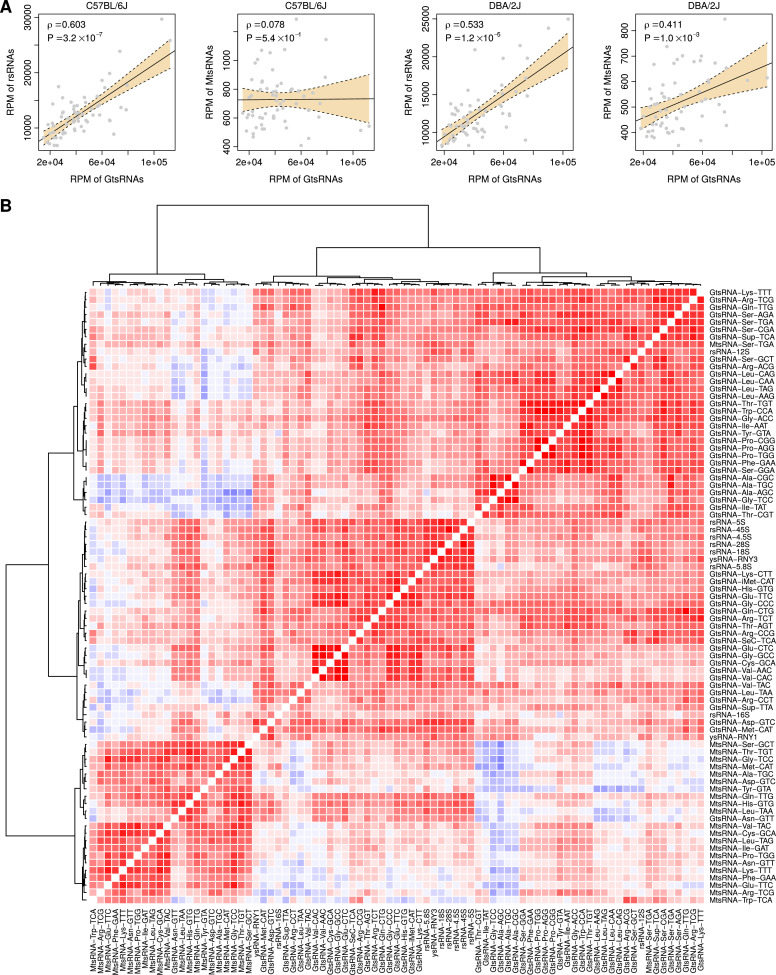
Comparison in co-expression pattern between the two mouse strains. (A) Correlation in total abundance between GtsRNA and rsRNA and between GtsRNA and MtsRNA. *Spearman*’s rank correlation test was used to measure the relationship between the individual sncRNA categories. (B) Co-expression heatmap of the noncanonical sncRNA families in the C57BL/6J strain. *Spearman*’s rank correlation test was used to measure the pairwise relationship in expression between the individual noncanonical sncRNA families. Red means positively co-expressed (positive correlation) while blue means negatively co-expressed (negative correlation).

Firstly, we investigated the relationship in total abundance between rsRNAs, GtsRNAs, MtsRNAs, and ysRNAs. Generally, significant positive correlation was identified between each other, except for the relationship between GtsRNAs and MtsRNAs in the C57BL/6J mice and between ysRNAs and MtsRNAs in both strains (Fig. 5A and Supplementary Table S4). Secondly, we investigated the individual ts/rs/ysRNA families and analyzed the co-expression pattern in both strains. Fig. 5B demonstrated that there was a co-expression cluster dominantly composed of MtsRNAs in the C57BL/6J mice while the co-expression between MtsRNAs and other sncRNAs was generally weaker. In contrast, MtsRNAs in the DBA/2J mice appeared to be positively co-expressed with more sncRNA families, including rsRNAs, GtsRNAs, and ysRNAs (Supplementary Fig. S2). All these results suggest that there is a strain-specific co-expression pattern for MtsRNAs between the C57BL/6J and DBA/2J mice.

### 3.5 Co-expression of noncanonical sncRNAs with miRNAs

We also performed a co-expression analysis to understand the relationship between noncanonical sncRNAs and well-studied miRNAs. For each mouse strain, only the miRNAs with mean RPM > 1 were considered here. *Spearman*’s rank correlation test was used to measure the relationship in expression for the individual ts/rs/ysRNA-miRNA pairs. A ts/rs/ysRNA-miRNA pair with adjusted *P* < 0.05 was deemed either positively or negatively co-expressed. Interestingly, the ts/rs/ysRNA-miRNA co-expression pattern appeared to be similar between the C57BL/6J and DBA/2J mice (Supplementary Fig. S3). In total, 1318 positively co-expressed pairs and 156 negatively co-expressed pairs were identified to be commonly co-expressed in both the C57BL/6J and DBA/2J mice (Supplementary Fig. S3 and Supplementary Table S5). Although the exact function of noncanonical sncRNAs in mouse lung remains largely unknown, these results suggest that some ts/rs/ysRNAs may directly or indirectly co-operate with miRNAs, but the functional principle may not be necessarily similar to the well-known RNAi-like mechanisms (Chen and Zhou, 2023).

## 4 Discussion

We analyzed the noncanonical sncRNA profile in the lung during IAV infection, comparing two inbred mouse strains known for their genetically determined variances in susceptibility to IAV. We demonstrated substantial differences in ts/rs/ysRNA populations between the two strains during IAV infection. The recombinant inbred BXD parental strains, C57BL/6J and DBA/2J, have been widely employed to investigate various genetic aspects of immunology and infectious disease due to their significant variations in immune response and susceptibility to pathogens (Li et al., 2021; Morokata et al., 1999; Tamiya et al., 2020). For instance, DBA/2J mice display more severe lung pathology, elevated levels of chemokines/cytokines and viral replication, and delayed viral clearance upon viral challenge compared to C57BL/6J mice (Li et al., 2021; Morokata et al., 1999). In our study, four sncRNA families, rsRNA-12S, GtsRNA-Arg-CCT, GtsRNA-Arg-TCT, and GtsRNA-Lys-TTT, were upregulated during IAV infection. This upregulation occurred earlier in the DBA/2J mice compared to the C57BL/6J mice.

IAV infection can cause notable changes in certain types of host sncRNAs types, some of which modulate the host's antiviral defenses, while others facilitate viral replication (Bamunuarachchi et al., 2023; Zheng et al., 2020; Zhuravlev et al., 2022). Nonetheless, our comprehension of the role of sncRNAs in the host during IAV infection remains restricted. Several methodologies have emerged to advance sncRNA-seq techniques, facilitating the discovery of previously unnoticed noncanonical sncRNAs (Cozen et al., 2015; Shi et al., 2021). Therefore, noncanonical sncRNAs have gained considerable attention from researchers. Recent research has revealed the presence of tsRNAs in various organisms, emphasizing their potential role in the development of different diseases (Chen et al., 2024; Soureas et al., 2024; Zhao et al., 2024). Additionally, rsRNAs were identified in diverse tissues and demonstrated a sensitive response to pathological conditions, including metabolic disorders and Alzheimer's disease (Hernandez et al., 2023; Zhang et al., 2020). Our study found that both strains share a large proportion of differentially expressed tsRNAs/rsRNAs/ysRNAs with a common response to IAV infection.

The impairment of mitochondria may influence the production of MtsRNAs, potentially modulating the outcome of an infection. For example, MtsRNAs expression is altered in many conditions, including cancer, bacterial infections, and aging (Li et al., 2024; Looney et al., 2021; Soureas et al., 2024). In cases of *mycobacterium tuberculosis* infection, disrupted mitochondrial membrane architecture facilitates interaction between mitochondrial tRNAs and tRNA-cleaving RNAses, increasing mitochondrial tRNAs production (Looney et al., 2021). Furthermore, the accumulation of mitochondrial Glu-5′tsRNA-CTC during aging impairs mitochondria translation, causing deficiencies in mitochondrial structure, glutamate levels, and age-related memory (Li et al., 2024). Our investigation revealed a distinct differential expression of MtsRNAs between C57BL/6J and DBA/2J mice during IAV infection. We also observed a strain-specific co-expression pattern of noncanonical sncRNAs between C57BL/6J and DBA/2J mice, especially regarding MtsRNAs. C57BL/6J mice demonstrated a comparatively weaker co-expression between MtsRNAs and other sncRNAs. Conversely, MtsRNAs exhibited positive co-expression with a broader range of sncRNA families, including rsRNAs, GtsRNAs, and ysRNAs in DBA/2J mice. These different mouse strains are associated with variations in viral replication, levels of pro-inflammatory cytokines, and IAV susceptibility (Alberts et al., 2010; Eisfeld et al., 2018). Upon infection with IAV, the susceptible DBA/2J strain exhibits a higher upregulation of immune response compared to C57BL/6J mice (Alberts et al., 2010; Srivastava et al., 2009). Furthermore, the mitochondrial dynamics might differ between C57BL/6J and DBA/2J mice in terms of basal mitochondrial metabolism, susceptibility to oxidative stress, and antioxidant capacity (Filiou et al., 2021; Huang et al., 2006). Nevertheless, the mechanisms underlying MtsRNAs regulation during IAV infection remain unclear. The extent of differential expression in MtsRNAs during IAV infection might be correlated with the duration of infection, the severity of mitochondrial distress, and susceptibility to IAV. Therefore, further research is needed to clarify this mechanism.

In this study, we observed a strong correlation of several host sncRNAs with IAV infection, showing different expression of ts/rs/ys RNAs in the host when comparing DBA/2J and C57BL/6J mouse strains. Our study revealed the differential expression of host sncRNAs following IAV infection. Further investigations are warranted to explore their functions and relationships, thus enhancing our understanding of the interaction between IAV and host sncRNAs. Also, it remains unclear whether the observed differential expression of noncanonical sncRNAs is specific to IAV infection or it's a common outcome upon infection of other viruses. Future research with other virus strains may provide insights into the virus-specific sncRNA expression in mouse lung.

## CRediT authorship contribution statement

**Eun-A Ko:** Writing – original draft, Funding acquisition, Formal analysis, Data curation, Conceptualization. **Tong Zhou:** Writing – original draft, Software, Methodology, Formal analysis, Data curation, Conceptualization. **Jae-Hong Ko:** Writing – review & editing, Writing – original draft, Project administration, Methodology, Funding acquisition, Conceptualization.

## Declaration of competing interest

The authors declare that they have no known competing financial interests or personal relationships that could have appeared to influence the work reported in this paper.

## Data Availability

None

## Appendix Supplementary materials

Supplementary Fig. S1. The number of differentially expressed MtsRNA species between mock treatment and IAV infection at different time points. Image, application 1

Supplementary Fig. S2. Co-expression heatmap of the noncanonical sncRNA families in the DBA/2J strain. *Spearman*’s rank correlation test was used to measure the pairwise relationship in expression between the individual noncanonical sncRNA families. Red means positively co-expressed (positive correlation) while blue means negatively co-expressed (negative correlation). Image, application 2

Supplementary Fig. S3. Comparison in ts/rs/ysRNA-miRNA co-expression pattern between the C57BL/6J and DBA/2J mice. Each dot represents one ts/rs/ysRNA-miRNA pair. *Spearman*’s rank correlation coefficient (*ρ*) was used to measure the relationship in expression for the individual ts/rs/ysRNA-miRNA pairs. The commonly co-expressed ts/rs/ysRNA-miRNA pairs in the C57BL/6J and DBA/2J mice were highlighted. The correlation in *ρ* between the two mouse strains was computed by *Spearman*’s rank correlation test. Image, application 3

Image, application 4
