# Supplementary figures and images for: Insight into noncanonical small noncoding RNAs in Influenza A virus infection
Source: Virus Res. 2024 Sep 27;350:199474. doi: 10.1016/j.virusres.2024.199474 (PMC11466576; doi:10.1016/j.virusres.2024.199474)

### Upregulated

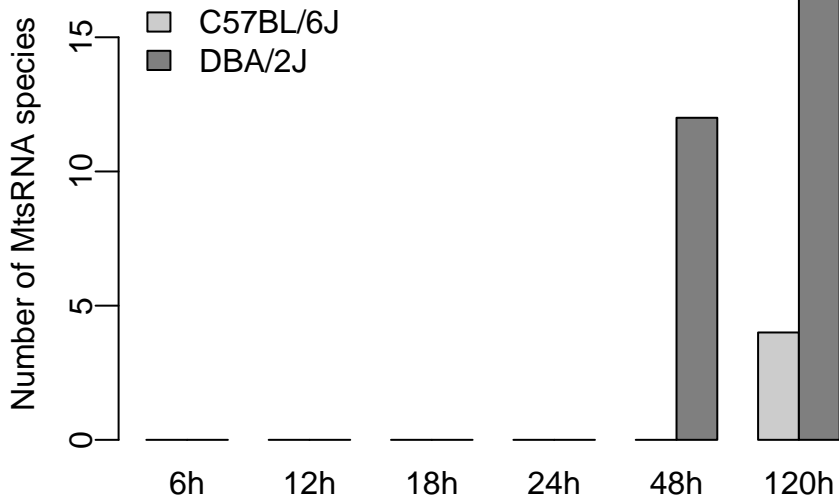

### Downregulated

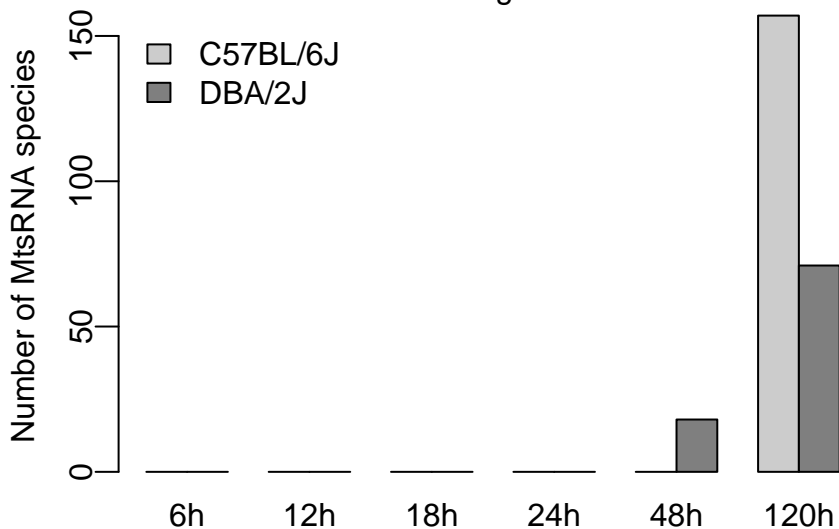

Supplement: Supplementary file 1 — Supplementary Fig. S1. The number of differentially expressed MtsRNA species between mock treatment and IAV infection at different time points. [file mmc1.pdf]

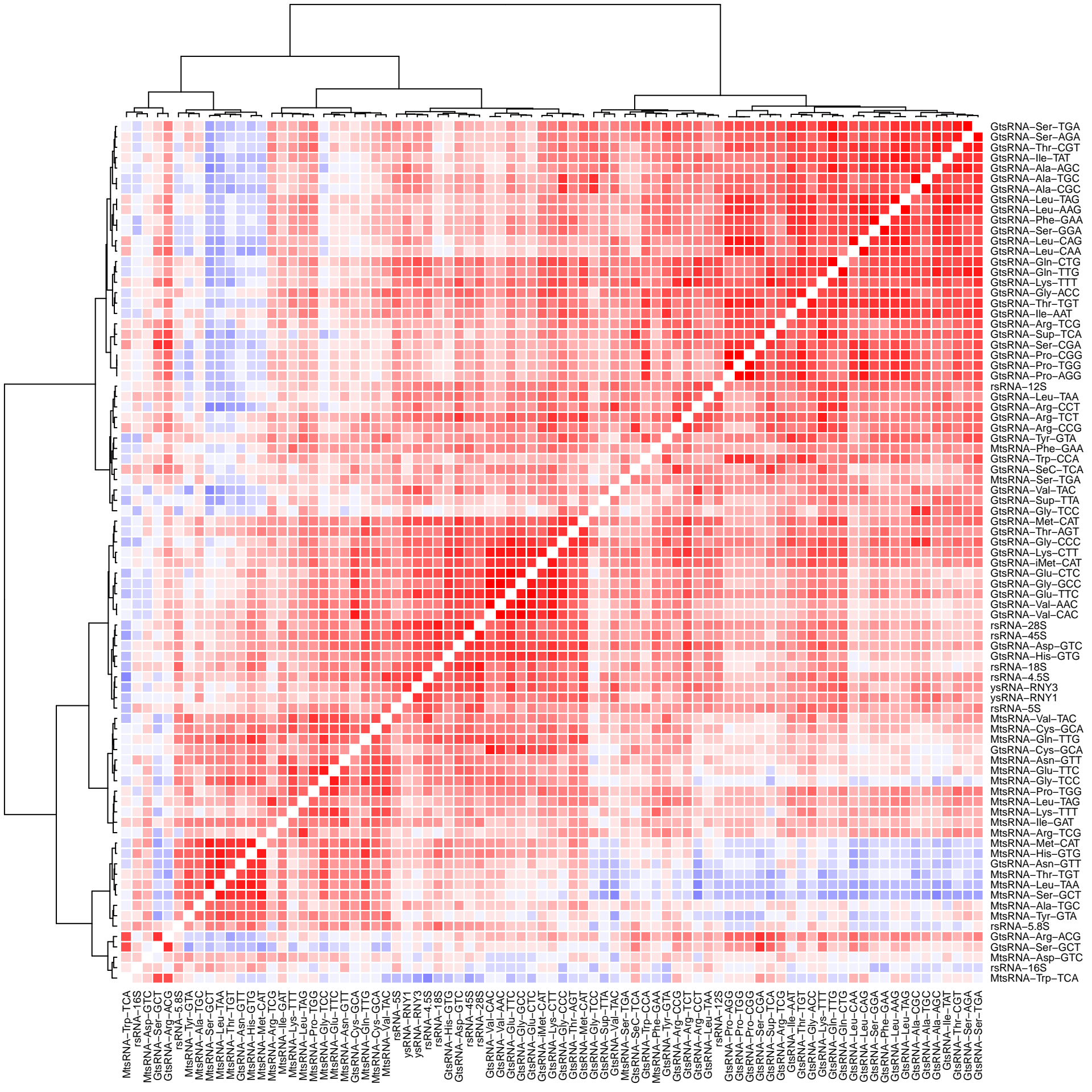

Supplement: Supplementary file 2 — Supplementary Fig. S2. Co-expression heatmap of the noncanonical sncRNA families in the DBA/2J strain. Spearman’s rank correlation test was used to measure the pairwise relationship in expression between the individual noncanonical sncRNA families. Red means positively co-expressed (positive correlation) while blue means negatively co-expressed (negative correlation). [file mmc2.pdf]

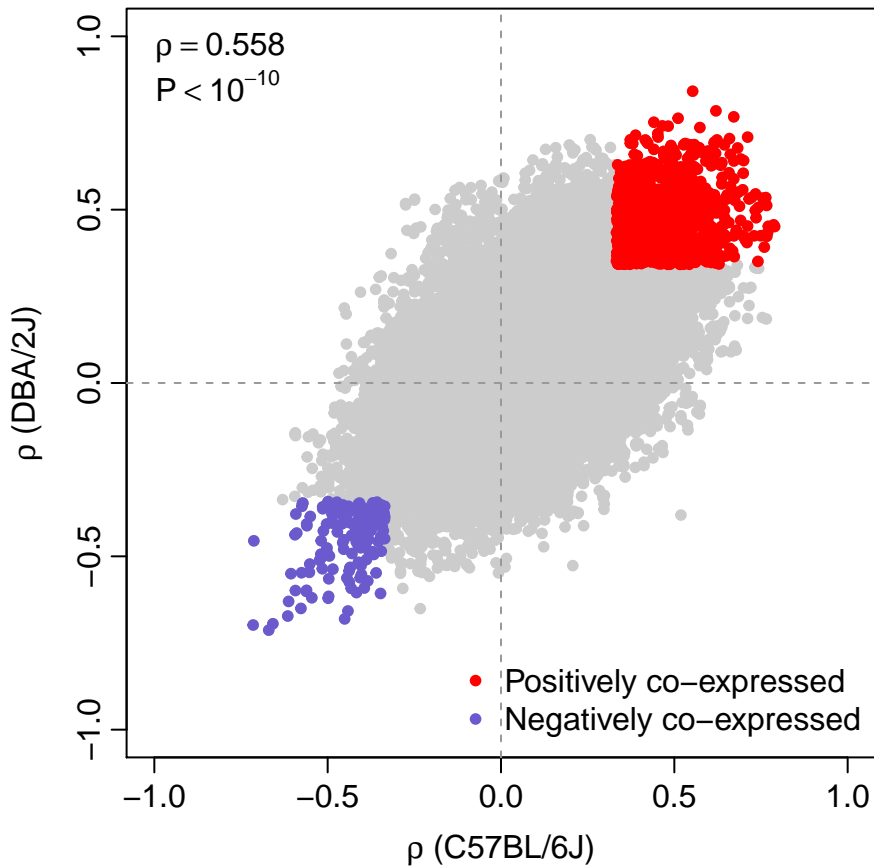

Supplement: Supplementary file 3 — Supplementary Fig. S3. Comparison in ts/rs/ysRNA-miRNA co-expression pattern between the C57BL/6J and DBA/2J mice. Each dot represents one ts/rs/ysRNA-miRNA pair. Spearman’s rank correlation coefficient (ρ) was used to measure the relationship in expression for the individual ts/rs/ysRNA-miRNA pairs. The commonly co-expressed ts/rs/ysRNA-miRNA pairs in the C57BL/6J and DBA/2J mice were highlighted. The correlation in ρ between the two mouse strains was computed by Spearman’s rank correlation test. [file mmc3.pdf]
